# The Microbial Metabolite Trimethylamine N-Oxide Links Vascular Dysfunctions and the Autoimmune Disease Rheumatoid Arthritis

**DOI:** 10.3390/nu11081821

**Published:** 2019-08-07

**Authors:** Marion M. Chan, Xiaofeng Yang, Hong Wang, Fatma Saaoud, Yu Sun, Dunne Fong

**Affiliations:** 1 Department of Microbiology and Immunology, Lewis Katz School of Medicine at Temple University, Philadelphia, PA 19140, USA; 2 Center for Inflammation, Translational and Clinical Lung Research, Lewis Katz School of Medicine at Temple University, Philadelphia, PA 19140, USA; 3 Center for Metabolic Disease Research, Lewis Katz School of Medicine at Temple University, Philadelphia, PA 19140, USA; 4 Department of Cell Biology and Neuroscience, Rutgers, the State University of New Jersey, Piscataway, NJ 08854, USA

**Keywords:** trimethylamine-*N*-oxide, cardiovascular disease, rheumatoid arthritis

## Abstract

Diet and microbiota each have a direct impact on many chronic, inflammatory, and metabolic diseases. As the field develops, a new perspective is emerging. The effects of diet may depend on the microbiota composition of the intestine. A diet that is rich in choline, red meat, dairy, or egg may promote the growth, or change the composition, of microbial species. The microbiota, in turn, may produce metabolites that increase the risk of cardiovascular disease. This article reviews our current understanding of the effects of the molecule trimethylamine-*N*-oxide (TMAO) obtained from food or produced by the microbiota. We review the mechanisms of actions of TMAO, and studies that associate it with cardiovascular and chronic kidney diseases. We introduce a novel concept: TMAO is one among a group of selective uremic toxins that may rise to high levels in the circulation or accumulate in various organs. Based on this information, we evaluate how TMAO may harm, by exacerbating inflammation, or may protect, by attenuating amyloid formation, in autoimmune diseases such as rheumatoid arthritis.

## 1. Introduction

Recent guidelines from the European League Against Rheumatism (EULAR) recommend aggressive management of cardiovascular disease (CVD) risk factors in addition to rheumatoid arthritis (RA) disease activity control, because severe subclinical atherosclerotic diseases underlie rheumatoid arthritis. Thirty to fifty percent of RA patients’ life expectancy is shorter than normal by 5–10 years, with heart attacks and strokes as the major causes of death. 

Diet has always been an essential factor in the management of atherosclerosis, RA, and other autoimmune diseases. More recently, the impact of dysbiosis, lack of diversity or imbalance of the intestinal microbiota has been recognized as a crucial factor in autoimmunity. Emerging from these two fields is the discovery that certain groups of intestinal bacteria process dietary compounds to produce metabolites that may benefit or compromise the host’s homeostasis [1]. One of the most notable paradigms is the conversion of choline and carnitine to trimethylamine N-oxide (TMAO), a molecule associated with inflammation and risk for many metabolic diseases. For example, TMAO promotes inflammation in adipose tissue of mice on a high-fat and high-sugar diet [2]. Excessively high levels of TMAO is an established risk factor for CVD [3,4,5,6], glucose intolerance [2], kidney damage [7,8,9], obesity, and diabetes [10,11,12]. High levels may also increase the risk of colorectal, hepatic, and prostate cancer in humans [13]. Large amounts of free trimethylamine (more than ten micrograms/mL) are excreted by patients with various degrees of hepatocellular failure [14]. 

## 2. Microbiome Alters the Dietary Composition

The human body, especially the intestine, is colonized by a vast amount of microbes. These intestinal bacteria, comprised of trillions of typically nonpathogenic commensal organisms, weigh nearly 0.2 kg [15,16,17]. The most abundant bacterial genera in the healthy gut are from the phylum Firmicutes, and within it, the most significant classes are the Gram-positive Clostridia and the Gram-negative Bacteroides. The Gram-negative Proteobacteria, with the Gram-positive Actinobacteria, Gram-negative Fusobacteria, and Verrucomicrobia, is also notable within the gut flora.

Human intestinal bacteria produce a variety of metabolites, some that benefit and others that compromise the host’s homeostasis [1]. The primary metabolites are branched-chain amino acids (BCAAs) and short-chain fatty acids (SCFAs). SCFAs are derived from bacterial fermentation of dietary carbohydrates and are used for energy and nutrients. The major SCFAs are acetate, propionate, and butyrate. Muscle uses acetic acid and the liver uses propionic acid to facilitate ATP production. Butyric acid provides energy to gut cells [18] and promotes the regulatory T lymphocytes, which calm the mucosal immune response, suppress inflammation, and maintain mucosal integrity in the colon [19]. Many enteric bacteria in the phyla Firmicutes, Bacteroidetes, and Actinobacteria, which encompass the probiotics in the orders of Bifidobacteriales and Lactobacillales, produce them [1]. BCAAs are derivatives from essential amino acids. *Clostridium sporogenes* metabolizes tryptophan to indole and subsequently to 3-indolepropionic acid, a highly potent neuroprotective antioxidant that beneficially scavenges hydroxyl radicals [19,20]. In contrast, *Prevotella copri* and *Bacteroides vulgatus* produce BCAAs that increase various health risks, for example, insulin resistance in humans [21].

TMAO is a microbiota-generated metabolite derived from choline and carnitine, which are essential nutrients contained in many foods, including red meat, eggs, and dairy. Choline has a wide range of biological activities; it maintains the structural integrity of cell membranes, supports cholinergic neurotransmission, and donates methyl groups in many biosynthetic reactions [22]. Carnitine transports long-chain fatty acids into the mitochondria to produce energy. The conversion of choline and carnitine to TMAO depends on the balance and diversity of the microbiota.

**Figure 1:**
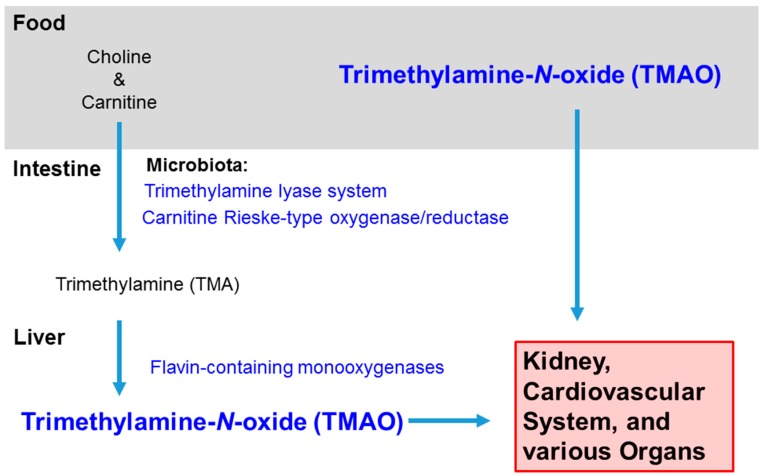
Sources and Distribution of Trimethylamine-N-oxide. TMAO intake can be direct, such as from fish, or indirect, converted from choline and carnitine in red meat, eggs, dairy, etc. In the intestine, bacteria in the microbiota convert choline and carnitine to trimethylamine (TMA), and then the hepatic enzymes flavin-containing monooxygenases further metabolize the molecule to trimethylamine-N-oxide. TMAO usually leaves the body in the urine, but it can accumulate in the kidney and rise to high levels in the circulation and various organs with inflammation.

In animal models, Chen et al. (2016) detected a higher number of *Prevotella* and a lower number of *Bacteroides*, *Lactobacillus*, and *Bifidobacterium* within the fecal content of mice fed choline [23]. Bacteria in the human gut possess the trimethylamine lyase system (CutC/D) and the carnitine Rieske-type oxygenase/reductase system (CntA/B and YeaW/X) for metabolizing choline and carnitine in the diet into trimethylamine (TMA). In the human liver, TMA is oxidized by flavin-containing monooxygenases (FMO) to TMAO [24,25] (Figure 1). Romano et al. (2015) identified by metabolomics eight distinct bacterial strains of TMA producers: *Anaerococcus hydrongenalis*, *Clostridium asparagiforme*, *Clostridium hathewayi*, *Clostridium sporogenes*, *Edwardsiella tarda*, *Escherichia fergusonii*, *Proteus penneri*, and *Providencia rettgeri* [26].

The levels of TMAO are also determined by the host intrinsic genetics. In humans, the plasma levels of TMAO correlate positively with an increase in age. TMAO is also higher in white participants according to a cross-nationality clinical trial that surveyed hyperparathyroid, diabetic patients on hemodialysis from 22 countries. Males with end-stage renal disease have significantly higher levels of TMAO than females even after adjusting for age and kidney function [27,28]. In NMRI and C57BL/6J mice, females have higher enzymatic activity in their hepatic tissue homogenates than males [26,29]. Bile acids and the action of the nuclear receptor FXR (NR1H4) mediate the expression of the *FMO* gene [29]. Several studies have demonstrated gender differences in the expression of the *FMO3* gene, due to hormonal effects, although the results are equivocal. Besides indirectly via microbial conversion, TMAO can also be acquired directly from certain foods, for example, from fishes. TMAO is naturally abundant in marine fish, especially deep-sea species [25,28,30,31]. It is essential to fish because the molecule stabilizes proteins against various destabilizing forces, depresses the freezing point of body fluids, and counteracts sinking [32,33,34,35,36,37,38]. The finding that levels of TMAO correlate positively with omega-3 fatty acids, mercury, and vitamin D corroborate the association with fish consumption [28]. 

TMA and TMAO distribute throughout the body and can accumulate in some tissues [39,40]. About half of the TMAO is excreted unchanged within a day through urine, sweat, and breath [39,41]. The remaining TMAO is reduced back to TMA by the bacterial enzyme TMAO reductase in the human gut [42]. The concentration of TMAO in blood plasma from healthy humans is around 3 μmol/L [43]. Healthy humans on a choline-rich or seafood diet may produce μmol/L to mmol/L of TMAO in their urine. The urine level of TMAO twenty-four hours after eating two egg yolks is 634 μmol, and four egg yolks is 944 μmol [44]. Fish can yield much higher levels. In healthy males, after consuming 8 ounces (227 g) of common seafood purchased from local stores, the urinary levels of TMAO and its precursor, TMA, reach 1000–8000 micromoles (μmol) in 8 h (3436 μmol for cod; 8230 μmol for halibut; 2614 μmol for mackerel; 2769 μmol for swordfish; 5648 μmol for squid) [45,46]. In experimental models, plasma concentration of TMAO is ∼0.6 μmol/L in rats [47], and <5 μmol/L in mice [48,49]. A two-week infusion of TMAO can bring their circulatory level to 60 μmol/L without any apparent toxicity [47].

In humans, TMAO levels can reach strikingly high levels, millimolar in urine and a tenth to hundredth of micromolar in plasma. Mitchell et al. (1999) detected 80–1840 μM trimethylamine (TMA) in the urine of 50% of patients with primary or secondary liver diseases [14]. In patients with fish odor syndrome, the amount of TMAO secreted in urine is about 40–50 mg a day [50]. The median plasma TMAO level was 94.4 μM in chronic kidney disease (CKD) patients, significantly higher than the 3.3 μM in the healthy individuals [27]. Diabetic patients with hyperparathyroidism who are on hemodialysis average higher than 56 μM, with 1103.1 μM at the maximum [51]. Mafune et al. (2016) proposed that impaired kidney function reduces the renal excretion of TMAO, which increases serum TMAO levels [52]. They showed that the plasma level could reach 14–53 μM in a cohort of Japanese patients suffering from CKD depending on the disease severity of renal function impairment [52,53]. Restoring the renal function by transplantation in five patients with CKD reduced levels of TMAO to those of controls. Therefore, TMAO is considered a uremic toxin, a compound that is usually filtered and excreted by the kidneys, but accumulates and exerts toxic effects on various systems when the renal function fails.

TMAO has beneficial as well as harmful effects. On the protective side, it serves as an osmolyte [6,36] that stabilizes protein structures against destabilizing forces, such as urea, and maintains the volume of intestinal cells under osmotic and hydrostatic stresses [34,37,54]. It supports oxidative phosphorylation as an electron acceptor for the Enterobacteriaceae flora that grows in the anaerobic intestine, thereby maintaining a healthy symbiotic balance in the microenvironment [29]. Contrary to its beneficial effects, TMAO is associated with many health hazards. Elevated TMAO levels have been linked with increased risk of cardiovascular events including myocardial infarction, recurrent stroke, and cardiovascular death as reviewed in [55]. Patients with 6–122 μM of TMAO in the plasma are about 3.4-fold more likely to have recurrent strokes or cardiovascular events within a year than those with plasma levels of <2.7 μM [3]. The five-year mortality rate among coronary artery disease patients who underwent elective nonurgent coronary angiography is 3.9-fold higher than normal (plasma levels of TMAO at <2.5 μM for healthy individuals versus >6.5 μM for patients) [56]. Experimental evidence corroborates with the clinical studies attesting to the relationship between TMAO and CVD. For example, high levels of TMAO, ingested directly or from bacterial metabolized choline, increase cardiac inflammation and severity of atherosclerosis in rats and apolipoprotein E-deficient mice [23,30,57]. Investigators have advocated using plasma levels of TMAO as a predictive marker for atherosclerosis, atrial fibrillation, and adipose dysfunction [58,59]. Nonetheless, Cho et al. (2017) eloquently noted that whether TMAO is a causative agent in disease development and progression in humans, or merely a marker of underlying pathology, remains inconclusive [60]. 

Epidemiological studies showed that renal failure enhances atherosclerosis and cardiovascular diseases and vice versa [61,62]. Even though the mechanism by which TMAO exacerbates kidney impairment remains preliminary, Tang et al. (2015) showed that chronically elevated plasma levels of TMAO, due to diet, can directly contribute to progressive renal fibrosis and dysfunction [63]. The escalation in TMAO levels correlates with an increase in systemic inflammation, as the rises in plasma IL-6, fibrinogen, and C-reactive protein have indicated [9]. An attractive hypothesis proposes that specific metabolites, TMAO, among the uremic toxins can be considered danger-associated molecular pattern (DAMP) molecules and homeostasis-associated molecular pattern (HAMP) molecules. These would activate pattern recognition receptors (PRRs), and the trained immune response of the innate lymphoid and myeloid cells in an antigen-independent manner [64,65,66,67]. This concept is consistent with the role of endothelial cells as participants of the trained innate immune responses [68]. Thus, TMAO probably escalates the cross-talk between arterioles and renal tubule inflammation. In apolipoprotein E-deficient mice, a model for atherosclerosis, the hypercholesterolemia leads to early renal dysfunction that can progress into CKD [62].

## 3. TMAO Promotes the Etiological Mechanisms of CVD in RA

TMAO exerts an impact on many major mechanisms in the atherosclerosis pathogenesis pathway. It promotes forward cholesterol transport and inhibits reverse cholesterol transport (RCT). It alters the function of macrophages, foam cells, endothelial cells, and the responsiveness of platelets [24].

Atherosclerosis initiates with hypercholesterolemia. TMAO interferes with the elimination of cholesterol. RCT is a crucial process that uses high-density lipoproteins (HDLs) to move excess cholesterol from peripheral tissues to the liver where the bile would convert the cholesterol into bile acids for excretion in the feces [24]. TMAO impedes this pathway by interrupting bile synthesis and metabolism [23,69]. TMAO downregulates two cytochrome P450 enzymes, cholesterol 7 a-hydroxylase (cyp7A1) that is rate-limiting for bile synthesis, and sterol 27-hydroxylase that participates in diverting cholesterol into bile acids. Hence, it decreases recirculation into the intestine for absorption [70]. Ding et al. (2018) showed that TMAO significantly lowers the total bile acid pool size in mice [69]. TMAO supplementation also markedly reduces the expression of two intestinal cholesterol transporters—Niemann-Pick C1 Like1 (Npc1L1), which transports cholesterol into enterocytes from the gut lumen, and ATP-binding cassette subfamily G members 5 and 8 (abcg5/8) which transport cholesterol from the hepatobiliary [71,72]. 

Elevated TMAO levels in serum affect the influx/efflux of fatty deposits on the artery wall and set the stage for atherosclerosis [73]. The accumulated cholesterol, carried on low-density lipoprotein (LDL) and very low-density lipoprotein (VLDL), penetrates the endothelium. Arterial macrophages infiltrate the subendothelial spaces to ingest the LDL and VLDL. Macrophages take up lipids with their scavenger receptors CD-36 and scavenger receptor A (SRA). Typically, they limit lipid accumulation, digesting the LDL into fatty acids, free or esterified cholesterol to excrete via the apolipoprotein A1 (ApoA1) component of HDL. TMAO, however, disrupts the homeostatic mechanisms by causing an imbalance in the uptake and efflux with the upregulation of CD-36 and SRA receptors [74]. In combination with impaired RCT by bile, cholesterol accumulates within the macrophages [3]. Many clinical studies have revealed a direct correlation between the plasma levels of TMAO with the burden and extent of coronary atherosclerotic plaque [22,75,76,77].

The fat-laden macrophages and smooth muscle cells become the foam cells that contribute to inflammation of the arteries and lead to atherosclerosis. They produce reactive oxygen species (ROS), proteases, chemokines, pro-inflammatory cytokines, and eicosanoids, leading to vessel dilation, damage, thrombosis, and hypoxia [3]. TMAO further enhances oxidative stress by inhibiting manganese superoxide dismutase 2 activation and sirtuin 3 expression, so the ROS are not effectively neutralized, as shown in human umbilical vein endothelial cells (HUVECs) [78,79]. In a redox-regulated manner, TMAO (30 µM) activates the nucleotide-binding oligomerization domain-like receptor family pyrin domain containing 3 (NLRP3) inflammasome, caspase-1 activity, and IL-1β and IL-18 production [79,80,81]. Boini et al. (2017) showed the ROS-thioredoxin-interacting protein (TXNIP)-NLRP3 pathway mediates the inflammatory actions of TMAO [80]. The ROS inhibitor *N*-acetylcysteine and mitochondrial ROS scavenger process can neutralize its effect [81,82].

Hypertension is a recognized cause of cardiovascular and CKD, with the latter being another significant TMAO health risk. The peptide hormone angiotensin is widely employed in blood pressure studies for it regulates blood pressure and promotes sodium retention by the kidneys. In a study with Sprague–Dawley rats, co-infusion of TMAO with angiotensin II induced hypertension at 100-fold above normal levels, even though TMAO did not affect blood pressure in normal, nonhypertensive animals. It sustained the arterial pressure in the angiotensin-induced hypertensive rats, prolonging it two-fold, from 5 days to 14 days. Both the systolic and diastolic blood pressure increased compared to the rats who had angiotensin infusion alone [47].

Endothelial dysfunction is an early event in atherosclerosis. Li et al. (2017; 2018) revealed the effect of TMAO on endothelial nitric oxide synthase (eNOS) using rats in a CKD model [83,84]. Normal 22-month-old rats had low levels of TMAO (14 µM) whereas those with CKD were 3–4 fold higher (50 µM), and their aorta had suppressed acetylcholine-dependent endothelium relaxation and eNOS activity. These suppressions are reversible by adding an inhibitor of trimethylamine formation, 3,3-dimethyl-1-butanol, in the drinking water [83,84]. In vitro, TMAO (30 µM) increases the loss in interendothelial tight junction integrity. A decrease in tight junction protein zonula occludens-1 (ZO-1) and an increase in dextran flux in cultured mouse carotid arterial endothelial cells reflect the increase in vascular permeability [80]. TMAO (100 µM) also downregulates the production of nitric oxide and increases the mRNA and protein expression of the intercellular adhesion molecule (ICAM) and the vascular cell adhesion protein (VCAM) in primary HUVECs [82,85]. 

**Figure 2:**
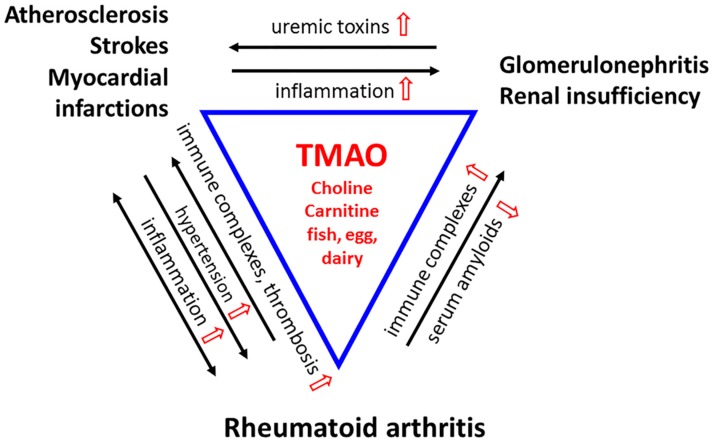
A Triad of Three Diseases. Rheumatoid arthritis (RA) is a chronic inflammatory disease in which immune complexes (RF and ACPA) induce inflammation in the microvasculature of the synovial joint. The stress is systemic. Cytokines produced locally disseminate into the circulatory system to cause inflammation in the arteries and veins. Chronic vascular inflammation leads to hypertension and hypoxia, which facilitates the development of RA-fibroblast-like synoviocytes (FLS) that invade the bone. Inflammatory stress leads to endothelial dysfunction, deactivation of endothelial nitric oxide synthase (eNOS), and compromised drainage. The increase in pro-inflammatory cytokines, chemokines, and adhesion molecules promotes the formation of atherosclerotic vascular lesions. These reactions feedback to the joints, exacerbating their inflammation and causing injury to the microvasculature. Platelets are hyperactivated, and unstable plagues increase the incidence of thrombosis, heart attacks, and strokes, the significant causes of death in RA patients. The immune complexes deposit in the glomeruli membrane, activating complement to damage the kidney. Uremic toxin levels rise throughout the circulation. Renal insufficiency promotes atherosclerosis through inflammation and vice versa. TMAO, derived from diet, heightens all these inflammatory reactions and leads to perpetuating cycles. Nonetheless, as a chemical chaperone and osmolyte, TMAO would protect proteins from the toxicity of urea, and acute phase proteins from aggregating into amyloids that compromise renal functions. (TMAO and its modulations are in red).

TMAO increases the incidence of thrombotic events, such as heart attacks and strokes [86]. Platelets move to the site of vascular injury to form plugs and enter a state of heightened reactivity. The activated platelets release various vasoactive compounds, clotting factors, and cytokines; then thrombosis, strokes, and myocardial infarctions occur. Evidence from humans and mice suggests that TMAO promotes platelet aggregation [86]. A study involving over 4000 subjects has shown that plasma levels of TMAO predict the incidence of thrombotic events [86]. Cheng et al. (2019) showed that TMAO promotes tissue factor activity in tumor necrosis factor-stimulated primary human coronary aortic endothelial cells. They also reported that the plasma TMAO level positively correlates with tissue factor activity in patients with ST-elevation myocardial infarction (STEMI) [87]. Animal models provide evidence that TMAO is causal to thrombosis [25]. Using antisense oligonucleotide targeting suppression and transgene, overexpression of the TMAO-generating enzyme, flavin-containing monooxygenase (FMO), Zhu et al. (2018) showed that plasma TMAO levels could modulate platelet activity [88] (Figure 2). 

Manor et al. (2018) elegantly validated the association between TMAO, microbiota, genetics, kidney function, and CVD in a large scale, multiomic, cross-sectional study with 648 individuals in a wellness program [28]. The study has verified the association of elevated levels of TMAO with protein-rich diets (Paleo and Low-Carbohydrate), the presence of metabolites from choline and carnitine and methane metabolism pathway (TMA is one of the substrates in methane metabolism).

Most significantly associated with TMAO levels are the kidney markers, decrease in glomerular filtration rate (GFR) and increase in blood urea nitrogen (BUN), consistent with the studies that have shown that elevated circulating TMAO can directly cause kidney damage. Other gut microbiota-derived metabolites that form uremic toxins are also high in the association. Among them are phenylacetylglutamine (PCG), indoxyl-sulfate, and p-cresol-sulfate (PCS), which are known indicators for glomerular sclerosis and interstitial fibrosis vascular disease and mortality in CKD patients. 

Plasma proteins associated with inflammation, CVD, and kidney disease also correlate with TMAO. For CVD, there are increases in growth/differentiation factor 15 (GDF-15), a predictor of mortality in stable coronary heart disease patients, in the galectin family, which are higher in the serum of atherosclerotic stroke patients, in chitotriosidase-1, which is implicated in the development of atherosclerosis, in P-selectin and IL-8, which are higher in unstable coronary heart disease patients, in CD40 ligand, which contributes to atherosclerosis, and caspase-3, which is expressed in atherosclerotic plaques. TMAO levels also positively correlate with junctional adhesion molecule-A (JAM-A), P-selectin, and matrix metalloprotease-1 (MMP-1), which induce platelet activation and enhance thrombus formation. Some proteins are associated with cardiovascular as well as kidney disease: adrenomedullin, kidney injury molecule 1, and TNF receptors, which negatively correlate with kidney function and predict cardiovascular disease in patients with CKD. 

## 4. Microbial Dysbiosis and TMAO Promote Rheumatoid Arthritis

In addition to metabolic diseases, microbial dysbiosis plays a role in the pathogenesis of autoimmune diseases both within and outside of the gastrointestinal (GI) tract [89]. Rheumatoid arthritis (RA) is an example of these autoimmune diseases. It affects 1.3 million Americans [90,91]. RA patients harbor the TMAO-producing anaerobic, Gram-negative Bacteriodetes, *Prevotella copri.* It is abundant in the periodontium, the lung, and the intestine, correlating with adverse cardiovascular events in RA patients [92,93]. Furthermore, it may alter the metabolism of the microbiota to reduce the effectiveness of the common disease-modifying antirheumatic drug (DMARD) methotrexate [94].

The most noticeable symptoms of RA are inflammation of the synovial tissues and erosion of cartilage and bone in the joints of hands and feet. Nonetheless, RA is a systemic disease with cardiovascular risk factors, which include hypertension, hyperlipidemia, vasculitis, atherosclerosis, and endothelial dysfunction. RA patients may harbor severe subclinical atherosclerotic diseases and have up to a three-fold increased risk of cardiovascular disease. Recent clinical studies discovered that atherosclerosis can lead to a shortened life expectancy in RA patients. Fatal heart attacks and strokes are the significant causes of mortality in these individuals [95,96,97]. Okano et al. (2017) and Olah et al. (2017) detected that carotid atherosclerosis plaque with distal occlusion in the middle cerebral artery (MCA) are more frequent and severe in RA patients [98,99]. The pronounced distal occlusions impair circulatory reserve capacity. CVD is a formidable risk in RA patients [100].

Collagen-induced arthritis (CIA) is one of the most widely accepted murine models for human RA. The DBA/1 strain of mice used in this model is uniquely susceptible to developing RA in response to immunization with type II collagen (CII) plus complete Freund’s adjuvant (CFA) [101,102,103,104]. Interestingly, these mice are also susceptibility to dyslipidemia when on an atherogenic diet (https://www.jax.org/strain/000670) [105,106]. After a high-fat diet for 14 weeks, there are both dyslipidemia and atherosclerotic lesions [107]. 

RA and atherosclerosis share many pathological mechanisms as extensively shown in humans and rodent models. Mice develop severe arthritis in joints from anti-collagen or anti-glucose-6-phosphate isomerase (G6PI) antibodies [102,108,109]. RA patients develop autoantibodies such as rheumatoid factor (IgM antibody to self IgG) and anti-citrullinated peptide antibody (ACPA) [110,111,112]. Citrullinated fibrinogen, citrullinated vimentin, and citrullinated histone H2B are present within both the atherosclerotic plaques and the arthritic joints of patients [113,114,115]. Whereas high titers of ACPA are in the circulation of RA patients [99], ACPA, such as anti-citrullinated vimentin, are in the calcified coronary arteries [99,116]. The citrullination and ACPAs could be the underlying mechanism for accelerated atherosclerosis and severe carotid atherosclerotic plaque in RA patients. This is because the autoantibodies form immune complexes that stimulate the inflammatory process and cause tissue damage via the complement system [117]. Continuous production of such immune complexes ultimately results in the chronic inflammation that is characteristic for RA [118] (Figure 2). 

Inflammatory and immune reactions are central to the pathogenesis of atherosclerosis and RA. Both diseases involve activation of mitogen-activated protein kinase (MAPK) and nuclear factor kappa-light-chain-enhancer of activated B cells (NF-κB), NLRP3-inflammasome, proinflammatory cytokines, and adhesion molecules [119,120,121,122,123,124,125,126,127]. TMAO upregulates the inflammatory pathways [125] and drives these signaling events in endothelial cells and leukocytes [74,128,129]. In RA, monocytes and neutrophils produce the proinflammatory cytokines tumor necrosis factor (TNF)-α and interleukin (IL)-1β within the locally affected joints, but they may enter the circulation to initiate inflammation of the blood vessels, affecting the arterial walls [122,130] (Figure 2). 

Macrophages transmigrate into the intima of vessel walls, causing the development of atherosclerosis as they accumulate in the joints, resulting in damaging the vasculature, tissues, bone, and cartilage [131]. Atherosclerosis develops with their actions on the endothelial cells. They activate the transcription factors NF-κB and activator protein 1 (AP-1) to produce monocyte chemoattractant protein-1 (MCP-1) and adhesion molecules such as ICAM-1 to promote phagocyte chemotaxis and infiltration [122]. Without IL-1, vascular inflammation and atherosclerosis do not develop in apolipoprotein E-deficient mice, and arthritis does not develop with collagen induction [132]. 

Dragoljevic et al. (2018) described monocytosis (Ly6-C^hi^ subset), leukocytosis, and prominent neutrophilia in the peripheral blood mononuclear cells from RA patients [131]. These cells have a significant reduction in ABCA1 and ABCG1, transporters that regulate cholesterol efflux [129]. They conducted a study on atherosclerotic lesion regression in the serum-induced murine models of RA efflux [131]. Female *Ldlr^−/−^* mice were placed on a Western type diet (WTD) for 14 weeks to initiate atherosclerosis. At this time point, a subset of mice was euthanized for baseline atherosclerotic lesion characterization, while the remaining mice were switched to a standard chow diet to reduce circulating cholesterol levels and initiate lesion regression. A week later, a subgroup of mice was rendered arthritic by CIA and the K/BxN serum transfer, while a set of mice remained untreated. Comparatively, the atherosclerotic lesions in the RA mice failed to regress. They were more extensive and laden with lipids and macrophages. The nonregressed lesions in the K/BxN serum transferred mice also revealed reticulated thrombocytosis and enhanced platelet-leukocyte interaction. 

The autoantibodies (RF and ACPA) and immune complexes from RA patients activate the platelets [133]. The plasma and synovial fluid of RA patients have elevated levels of platelets and platelet microparticles that bind to endothelial cells and feed the inflammatory responses, as do the cytokines. Patients with RA are 2–3-fold more likely to develop deep vein thrombosis or peripheral thromboembolism and 1.3-fold higher incidence of acute myocardial infarction [134,135]. Sudden deaths from pulmonary embolism also occur in RA patients [136] (Figure 2).

Table 1 summarizes the pathophysiological mechanisms shared by RA and CVD and the actions of TMAO on these mechanisms. The primary disease-causing culprits in RA and CVD are different. Autoantibodies initiate RA, whereas oxidized LDL initiates atherosclerosis. Both agents induce chronic activation of macrophages and endothelial cells. MAPK kinases and NFκB turn on the production of inflammatory cytokines, such as IL-6 and TNF, chemokines, such as IL-8, and adhesion molecules, such as ICAM and VCAM. The NLRP-3 inflammasome activates IL-1. The activated monocytes/macrophages release proteases and reactive free radicals, causing damage in the intima of the blood vessels or synovial lining of the joints. Macrophages, dendritic cells, T cells, and B cells accumulate at the sites of injury. Dying and dead cells accumulate. At the endothelium, loss of eNOS leads to endothelial dysfunction, hypertension, and hypoxia. Decreases in ZO-1 dilates tight junctions, leading to edema in the joints, and increases in tissue factor trigger thrombosis, leading to myocardial infarction. 

TMAO, from seafood or intestinal microbes, escalates systemic inflammation, vascular dysfunction, atherosclerosis, platelet hyperactivity, and the associated pathological conditions. It is therefore likely to aggravate autoimmune diseases, as dysbiosis, leaky intestinal barrier, microbial metabolites are significant factors. 

## 5. TMAO Can Harm or Protect the Renal Function of RA Patients

RA patients, as well as many who suffer from other rheumatic diseases, are at risk for renal insufficiency [137,138,139]. Approximately 15–25% of RA patients have reduced glomerular filtration rate (GFR) <60 mL/min [140], even though the frequency of end-stage renal disease in patients with RA is 1% [138]. The nephrotoxicity of therapeutic drugs, such as NSAIDs, d-penicillamine, and gold, are among the culprits that cause proteinuria and the concurrent renal disease that significantly increase mortality in RA patients.

Even though TMAO and its probable signaling mechanisms are associated with CVD and CKD, a contention exists regarding whether it would harm or protect the renal system. In RA, IL-6 activates the production of acute phase proteins, which can aggregate into toxic insoluble serum amyloid A. The amyloids deposit in the glomeruli, blood vessels, peritubular, and interstitium, resulting in kidney damage. CKD patients classically present with proteinuria, increase in serum creatinine level, and then progressive renal failure. Renal biopsies from RA patients with a clinical renal disease showed that 30% have amyloidosis, and 17% have membranous glomerulonephritis [137]. 

TMAO may critically benefit renal function in systemic inflammatory diseases by being a “chemical chaperone” to enhance the folding and stability of proteins, and an osmolyte to maintain cellular fluid balance [6,36]. It accumulates in the endoplasmic reticulum to promote protein folding, thereby inhibiting ER stress and attenuating the formation of intracellular aggregates [141]. In the kidneys, accumulated TMAO counteracts the destabilizing effects of urea on macromolecular structures of proteins and nucleic acids, and offsets the inhibitory effects of urea on functions such as ligand binding [141]. 

The amyloids can also accumulate and aggregate in other tissues and organs, causing cardiomyopathy, hypertension, and corneal and vitreous abnormalities. TMAO protects epidermolysis bullosa simplex keratinocytes from heat stress-induced keratin aggregation. This suggests a therapeutic potential for TMAO in epidermolysis bullosa and other keratinopathies [142]. TMAO modulates binding of protein kinase A-phosphorylated tau protein to tubulin, implicating a therapeutic application in Alzheimer’s disease [36,143]. There is also a study that shows a moderate increase in plasma TMAO does not harm the circulatory system. In contrast, increased dietary TMAO seems to reduce diastolic dysfunction in the pressure-overloaded heart in spontaneously hypertensive rats. Therefore, TMAO may be renal protective in RA patients (Figure 2). 

## 6. Microbiota Remodeling Reverses TMAO-Mediated Pathologies

Although TMAO at high levels poses risks for metabolic and autoimmune diseases in humans, many studies have shown that its levels may be modulated via remodeling the gut microbiota. For example, consumption of phytochemicals, such as allicin in garlic and resveratrol in red wine and grapes, may change the composition of the gut microbiota and, in association, reduce the effect of TMAO. Wu et al. (2015) examined the effect of allicin (50 mg) on carnitine diet using C57Bl/6 mice [144]. Oral feeding of allicin reduced the carnitine-induced plasma level of TMAO to that of standard chow, concomitant with a change in the taxa of the gut microbiota. Similarly, Chen et al. (2016) have observed remodeling and inhibition of TMAO synthesis in C57BL/6 mice and the apolipoprotein E-deficient mice fed choline and treated with resveratrol [23]. When on a choline diet, the mice had an increased number of *Prevotella*, a decreased number of *Lactobacillus* and *Bifidobacteria*, and increased TMAO levels. Upon treatment with resveratrol (0.4% of chow), the microbial population levels reversed and the plasma TMAO levels decreased. 

Correspondingly, the treatment abolished the TMAO-induced atherosclerotic lesion area and the cholesterol content in the whole aorta of the apolipoprotein E-deficient mice. The investigators also observed an increase of CYP7A1 expression in the liver and an enhancement of fecal bile acid excretion. Thus, they attributed the atherosclerosis protective capacity of resveratrol to its effect on TMAO. Reduction of TMAO production may be contributing to the long regarded CVD protective effect of garlic and red wine.

Bresciani et al. (2018) reported that TMA production is lower from choline and L-carnitine when a vegetarian fecal starter, rather than omnivorous starter, is used for in vitro fermentation [145]. Furthermore, fecal starter from individuals consuming pomegranate juice and blood orange juice shows significant reduction of TMA production. Qiu et al. (2017) used *Enterobacter aerogenes* ZDY0 as a probiotic to reduce TMA in the gut, leading to reduced TMAO in the serum of the choline-fed mice [146].

## 7. Conclusions

Humans and microbes live in symbiosis. Bacterial species benefit humans by producing metabolites essential for humans. On the other hand, dysbiosis promotes bacterial species that may metabolize dietary components, such as choline and carnitine, into molecules that produce potential health risks. TMAO is recognized as a risk factor for CVD and could be a diagnostic marker because of the strong association between the two. TMAO interferes with cholesterol metabolism and induces inflammation. Atherosclerosis and chronic inflammation underlie many autoimmune diseases. RA patients, for example, have high mortality from stroke and heart attack. Eggs and dairy are rich in choline, which metabolizes to TMAO. Seafood naturally contains high levels of TMAO because it is vital to the physiology of marine fishes and crustaceans. Fishes, especially cold-water species such as salmon, tuna, sardines, and herring, have long been advised for patients to attenuate atherosclerosis and RA. Consumption of about two servings of marine fish can raise the urinary excreted TMAO to millimolar levels. Since fish contains omega 3-fatty acids as well as very high levels of TMAO, there is a need to re-evaluate whether increased consumption is protective of rheumatoid arthritis and CVD. TMAO also bears benefits. It stabilizes protein structure and cell volume. It is crucial to study the actions of TMAO to fully understand the epidemiological studies that show the benefits of seafood. 

## Figures and Tables

**Table 1:** Actions Common to TMAO and the Pathogenesis of RA and CVD.

Mechanism of Actions	TMAO	CVD	RA
Microbial dysbiosis	+	+	+
Genetics: Major histocompatibility molecules		+	+
Activated peptidyl arginine deaminase-4 (PAD-4)			+
Activated B cells		?	+
Induced production of anti-citrullinated peptide antibodies			+
Activated complement systems		?	+
Activated T cells		+	+
Induced production of Interluekin-17		+	+
Disrupted reverse cholesterol transport (RCT)	+	+	
Interrupted bile synthesis and metabolism	+	+	
Disrupted cholesterol uptake and efflux balance	+	+	
Upregulated scavenger receptor A1		+	
Activated macrophages in synovial lining or intimal layer	+	+	+
Activated mitogen-activated protein kinase (MAPK)	+	+	+
Activated Nuclear factor kappa-light-chain-enhancer of activated B cells (NF-κB)	+	+	+
Activated NLRP3 inflammasome	+	+	+
Activated caspase-1	+	+	+
Induced proinflammatory cytokines IL-1β, tumor necrosis factor-α, and interleukin-6	+	+	+
Released reactive oxygen species (ROS)	+	+	+
Production of chemokines	+	+	+
Liberation of eicosanoids		+	+
Upregulated cell adhesion molecules	+	+	+
Hypertension	+	+	+
Endothelial dysfunction: Inactivated endothelial nitric oxide synthase	+	+	+
Increased vascular permeability	+	+	+
Activated platelets and increased thrombotic events	+	+	+
Increased incidences of myocardial infarction and strokes	+	+	+

Abbreviations: TMAO, trimethylamine-N-oxide; CVD, cardiovascular disease; RA, rheumatoid arthritis; Notations: + indicates the observed effect; ? indicates exhibition of both detrimental and protective effects.
